# A scoring method to standardize lesion monitoring following intra-dermal infection of *Leishmania* parasites in the murine ear

**DOI:** 10.3389/fcimb.2014.00067

**Published:** 2014-05-28

**Authors:** Steffen Schuster, Mary-Anne Hartley, Fabienne Tacchini-Cottier, Catherine Ronet

**Affiliations:** ^1^Department of Biochemistry, University of LausanneEpalinges, Switzerland; ^2^WHO-IRTC CenterEpalinges, Switzerland

**Keywords:** *Leishmania*, murine model, ear infection, score, parasites

For several decades the murine experimental model of *Leishmania* infection has been invaluable in deciphering events occurring during the innate immune response and those involved in the differentiation of CD4^+^ T helper cells. Initially, most studies inoculated mice subcutaneously into the hind footpad with a high dose of stationary phase parasites (1 to 3 × 10^6^). In recent years, new protocols have been adopted to better mimic the natural transmission of the parasite caused by the bite of infected sand flies. To this end, low numbers of parasites (10–1000 metacyclic promastigotes) are injected intradermally in the ear. However, given the different anatomy of the ear compared to the footpad, monitoring lesion development in the ear is technically more difficult and until now there has been no consensus on how to monitor lesion progression in this anatomic region. The various methods, which are currently used, are listed below:

1. **Diameter**: The first reported and also most commonly used technique to measure lesional expansion, is by monitoring the induration diameter (Mitchell, 1983; Belkaid et al., 1998, 2000).
2. **Volume**: Lesional volume can be measured in three dimensions and reported as ellipsoids according to the following equation [(a/2 × b/2 × c/2) × 4/3π] (Maurer et al., 2006).
3. **Thickness**: Swelling across the transverse plane of the pinna can be measured using a caliper. Considering the narrowness of this dimension in a normal ear, thickness is technically difficult to quantify (Rosas et al., 2005; de Moura et al., 2007).
4. **Score**: To our knowledge, only one group has developed a scoring system, which is based on lesion diameter. This technique classifies 4 categories of disease severity ranging from small, localized swellings (scored 1) to advanced lesions larger than 6 mm in diameter (score 4) (Baldwin et al., 2007).
5. **Surface**: A more sophisticated approach requires bioluminescent parasites, through which *in vivo* imaging is able to delimit and quantify the area of active infection (Lecoeur et al., 2007).

In order to better evaluate these measurement systems, we compared them across various murine models known for their diverse disease outcome in *Leishmania major* infection. To this end, BALB/c, C57BL/6 and MyD88^−/−^ mice were inoculated intradermally in the central ear pinna with infectious promastigotes of *L. major* LV39. The 10 μl inoculations were either given at a high dose (10^5^ stationary-phase parasites) or a low dose (10^3^ metacyclic promastigotes). For high dose inoculation, no significant differences in lesion development were observed between stationary-phase or purified metacyclic promastigotes (data not shown). The evolution of lesion development was then analyzed through previously described methods and used to develop a more robust scoring system as presented below (Figure 1).

**Figure 1:**
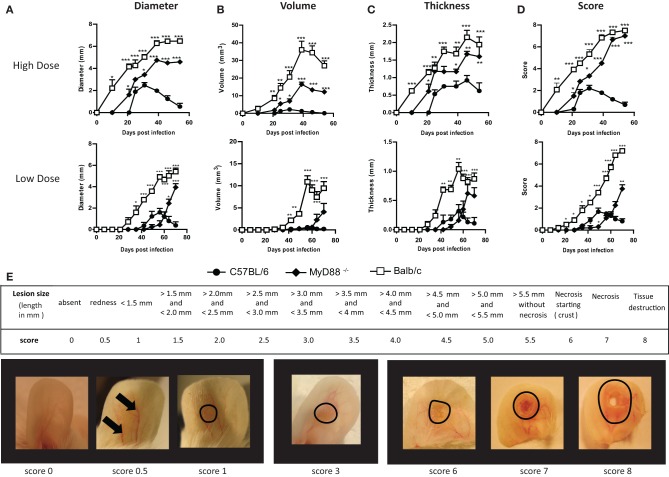
**A new scoring system to monitor lesion development of cutaneous leishmaniasis following *Leishmania* infection in the murine ear dermis**. C57BL/6, MyD88^−/−^ and BALB/c mice were inoculated intradermally in the central ear pinna with 10 μl of 10^5^ stationary phase *Leishmania major* promastigotes (high dose) or 10^3^ metacyclic promastigotes (low dose). Throughout the infection, lesion size was monitored and the results were expressed using the different methods: **(A)** Diameter; **(B)** Volume; **(C)** Thickness and **(D)** with our newly proposed scoring system. Below, a table describing this scoring method is given **(E)**. Pictures of BALB/c infected ears representing selected lesion scores of lesion throughout the infection are presented. Data are representative of at least two independent experiments with five mice per group (females, 5–8 weeks old). Results shown are mean + s.e.m. Statistical comparison to C57BL/6 were performed using the Student's *t*-test ^*^P < 0.05, ^**^P < 0.01, ^***^P < 0.001.

- **Diameter**: Among the techniques previously described, measuring lesion diameter proves to be most precise and representative of what is observed visually. However, major limitations of this technique become apparent at extremes in the infection time course. For instance, early events like inflammation and the appearance of a non-measurable papule are overlooked during the first weeks of infection, as seen in high dose infected C57BL/6 mice and low dose infected BALB/c mice (Figure 1A). Further, late events like necrosis and tissue destruction are not evaluated at all. Indeed, because there is a plateau in lesion diameter during late infection of susceptible mice (BALB/c and MyD88^−/−^), this method gives the impression that the disease is stabilizing when, in fact, the lesions are still progressing with the appearance of necrotic skin damage (Figure 1A).
- **Volume**: Lesion enlargement can also be measured by determining the progression of its volume. This method is better suited to monitoring large lesions in susceptible mice such as BALB/c and MyD88^−/−^ mice, as lesion development in *Leishmania*-resistant mouse strains, such as C57BL/6 mice, is reduced to a minor or nearly nonexistent phenotype (Figure 1B). Nevertheless, limitations are still observed when monitoring these larger lesions at later time points, where ulcerations, necrosis and atrophy degrade the lesional volume making them appear to become smaller in BALB/c and to a lesser extent also in MyD88^−/−^ mice (Figure 1B).
- **Thickness**: We found the measurement of lesional thickness to be the least precise technique to follow cutaneous leishmaniasis in murine ear infection (Figure 1C). Moreover, the technical difficulty of this method resulted in a wide variability between different studies as well as between investigators. In addition, differences between susceptible and resistant mice are reduced (Figure 1C), making analysis of slight modulation between groups more difficult. Most importantly, the appearance of necrosis and tissue loss is also not considered.

In conclusion, we found that all of the previously described techniques for monitoring auricular cutaneous leishmaniasis in a murine model, did not accurately measure lesion development over the full course of disease or failed to represent what is observed visually. Limitations were particularly evident at extremes of the infection time course, where events such as the first signs of inflammation and end-stage necrosis are not considered. For this reason, we propose to introduce a new scoring system. This system will take into account each step of lesion evolution occurring during the infection: from the first signs of lesional inflammation such as erythema to the development of necrosis with tissue loss (Figure 1E). Further, we propose it as suitable for both low and high dose infections.

Briefly, lesion scores range between 0 and 8 (Figure 1E). At the onset of infection, the first visible sign of infection is erythema (redness due to capillary swelling) and is assigned a score of 0.5. The appearance of palpable swelling or a papule receives a score of 1. As soon as borders are discernable, lesional size is quantified by measurement of the length and breadth using a caliper. The longer dimension (length) is used to assign a score for each increment of 0.5 mm. For example, a length between 2.00 and 2.49 mm is awarded a score of 2, while the next interval (2.50–2.99 mm) is given a score of 2.5, and so on until a maximum value of 5.50 mm (score 5.5). For lesions that have a diameter higher than 5.50 mm but do not show any signs of necrosis, a score of 5.5 is attributed. The first evidence of necrosis is gauged by an increase of 0.5 points (score 6). Severe necrotic lesions without tissue loss receive a score of 7 and once tissue destruction at the site of infection appears, a score of 8 is attributed (Figure 1E). When a score of 7–8 is reached, mice are sacrificed for ethical reasons.

Consequently, this scoring system depicts the evolution of lesion development in a precise manner that accurately represents the phenotypic progression of disease (Figure 1D). Importantly, small modulations in lesion onset and evolution are also discernable, especially at the beginning of lesion formation and at later time points when tissue modifications and necrosis occur. Besides being a useful method of quantifying disease, a representative picture would be informative, especially at the onset of necrosis.

This method can be used independently of the number of parasites inoculated and we also propose it for monitoring “natural” infection models, where small numbers of parasites are transmitted by an infected sand fly. While this study clearly shows the limitations of using single measurements in quantifying disease progression for high-dose infections, it also shows that low-dose infections may be accurately monitored by lesional diameter, as this parameter closely mirrored that of the score. An advantage of our system however, is the possibility to present lesion evolution from mice resistant or susceptible to *Leishmania* infection on the same graph, without the bias created by the under-representation of very small and very large lesions that was observed in the other techniques. Finally, since the presented method is technically easy to apply it will allow comparison and standardization between the increasing number of studies performed using intradermal inoculation of *Leishmania* in both basic and translational research. This should help evaluating novel vaccine candidates as well as drug screening in murine experimental *Leishmania* infection models in the ear.

## Conflict of interest statement

The authors declare that the research was conducted in the absence of any commercial or financial relationships that could be construed as a potential conflict of interest.
